# A case report of thoracic SMARCA4-deficient undifferentiated tumor associated with brain metastases

**DOI:** 10.3389/fonc.2026.1718235

**Published:** 2026-04-22

**Authors:** Min Yang, Yang Wang, Xiaolong Fang, Yuntao Wang, Qingliang Xu, Chunyan Tang, Li Qi, Aijie Li, Jingjing Li

**Affiliations:** 1 Affiliated Hospital of Shandong Second Medical University, Weifang, Shandong, China; 2 Jinming Yu Academician Workstation of Oncology, Shandong Second Medical University, Shandong, China; 3 Department of Thyroid and Breast Surgery, Affiliated Hospital of Shandong Second Medical University, School of Clinical Medicine, Shandong Second Medical University, Weifang, Shandong, China

**Keywords:** clinical trials, immunotherapy, SMARCA4 deficiency, SMARCA4-UT, treatment

## Abstract

Thoracic SMARCA4-deficient undifferentiated tumors (SMARCA4-UT) were considered distinct from the traditional NSCLC with SMARCA4-deficient (SMARCA4-dNSCLC) in the 5th edition of the WHO classification of thoracic tumors in 2021. Due to the low incidence rate and the lack of sufficient research, clear treatment guidelines for SMARCA4-UT are currently lacking. We report a rare case of thoracic SMARCA4-deficient undifferentiated tumor with multiple brain metastases. Following treatment with cranial metastasis radiotherapy and systemic immunotherapy combined with chemotherapy, the lesions significantly decreased in size, and the patient’s symptoms improved markedly. This article retrospectively analyzed the diagnosis and treatment process of a case of SMARCA4-UT and discussed therapeutic progress and future prospects. Given the limitations of current single-drug therapy, the combined use of immunotherapy, chemotherapy, and new target drugs is an essential choice and the future direction.

## Introduction

Approximately 5%-10% of lung cancers have a SMARCA4 deficiency mutation. About 5% of conventional non-small cell lung cancer (NSCLC) cases lack SMARCA4 (1). However, thoracic SMARCA4-deficient undifferentiated tumors (SMARCA4-UT) were considered distinct from the traditional NSCLC with SMARCA4-deficient (SMARCA4-dNSCLC) in the 5th edition of the WHO classification of thoracic tumors in 2021 (2). SMARCA4-UT mainly invades the mediastinum and lung parenchyma, forms large infiltrative masses, and compresses the surrounding tissues (3). Due to the low incidence rate and the lack of sufficient research, clear treatment guidelines for SMARCA4-UT are currently lacking. This article retrospectively analyzed the diagnosis and treatment process of a case of SMARCA4-UT at the Affiliated Hospital of Shandong Second Medical University, and discussed therapeutic progress and future prospects.

## Case report

### Patient information

The patient is a 68-year-old male retiree. He reports a 20-day history of headache accompanied by blurred vision and unsteady gait. Past medical history includes coronary artery disease and coronary stent implantation. He has been taking aspirin, clopidogrel, and rosuvastatin for several years. No known allergies. He has a 50-year smoking history at 30 cigarettes per day and a 50-year drinking history at 35 grams of pure alcohol daily, equivalent to 3.5 standard drinks. No relevant family history.

### Clinical findings

**Figure 1:**
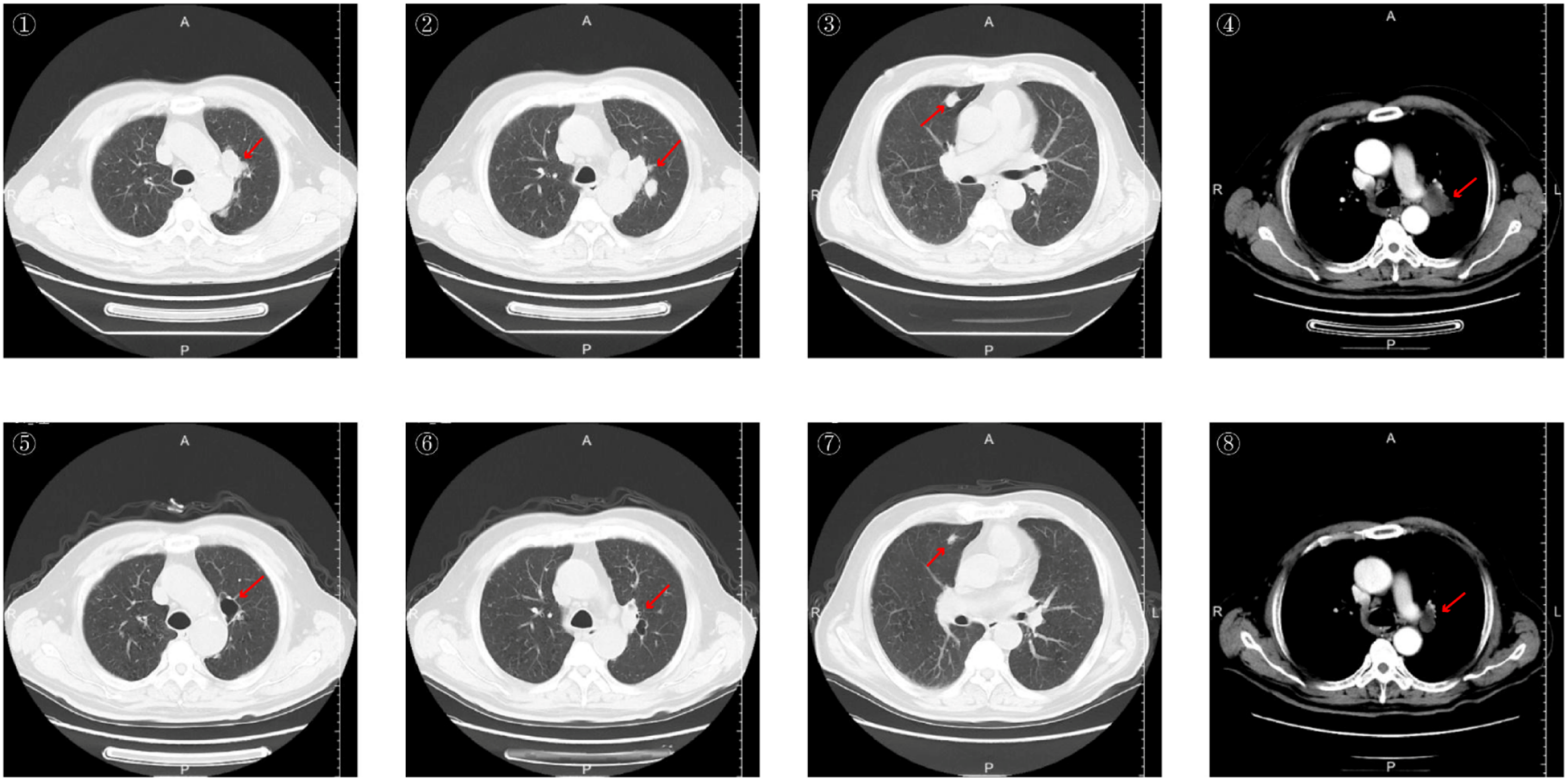
Chest CT images before and after treatment. Images ①②③④ show pre-treatment chest CT scans taken on July 17, 2025, revealing a mass at the hilum and within the left lung. Images ⑤⑥⑦⑧ display post-treatment chest CT scans taken on October 29, 2025, indicating tumor shrinkage compared to previous scans, with partial cavitation observed.

**Figure 2:**
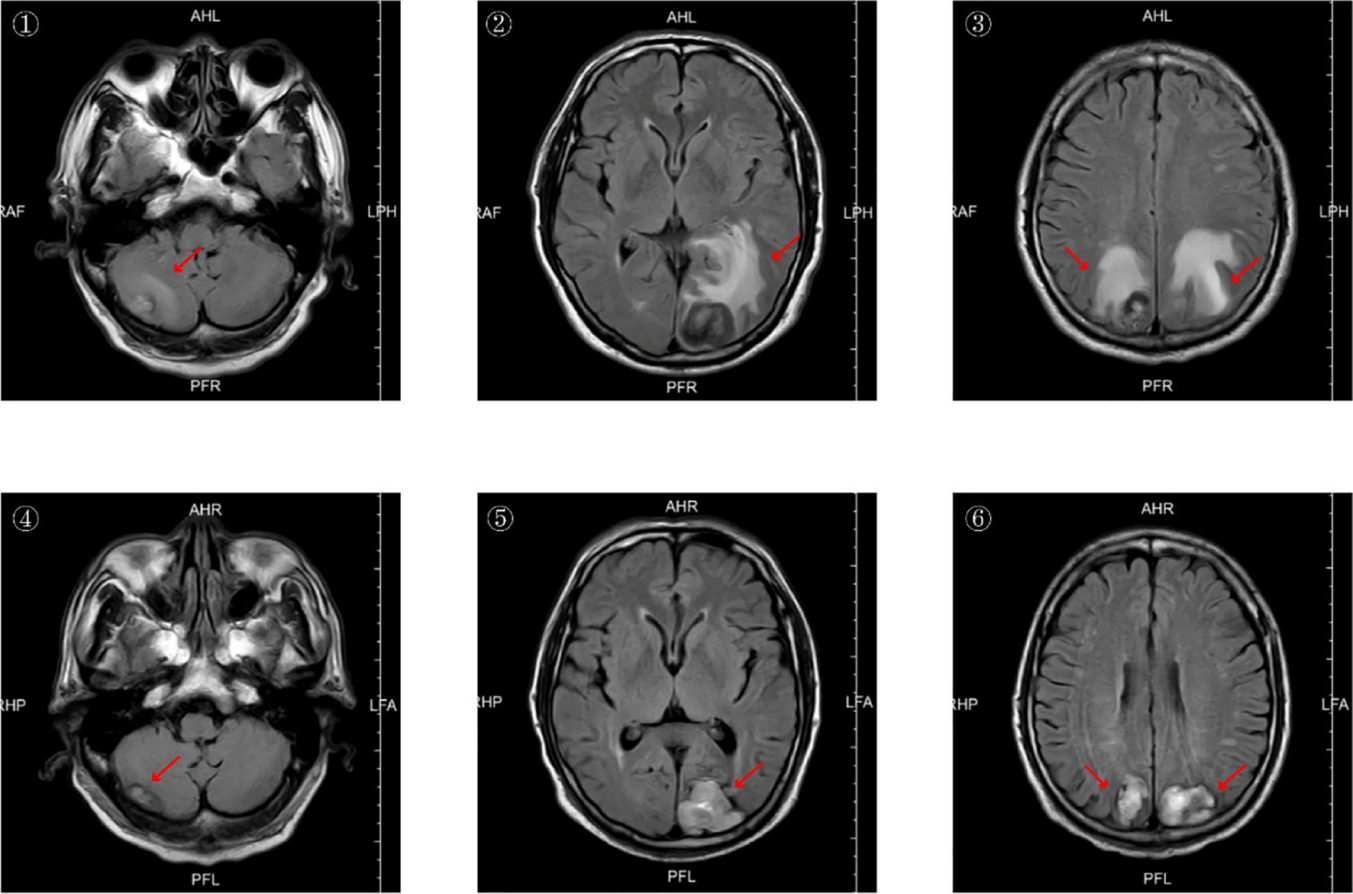
Pre- and post-treatment cranial MRI scans. Figures ①②③ show pre-treatment cranial MRI on July 16, 2025, revealing bilateral parieto-occipital and right cerebellar masses accompanied by significant edema. Figures ④⑤⑥ show cranial MRI scans taken on October 30, 2025, after treatment. The original edema has resolved, and the cranial metastatic tumors have also decreased in size compared to the previous scans.

The patient is a 68-year-old male with a 20-day history of headache, as detailed in the patient information. Upon admission, the patient’s vital signs—temperature, heart rate, respiration, and blood pressure—were all within normal ranges. Physical examination revealed decreased visual acuity and unsteady gait. ECOG performance status was 2. Chest CT reveals possible lung cancer in the left lung with multiple intrapulmonary metastases and enlarged mediastinal lymph nodes. (**Figure 1**) Cranial MRI shows multiple tumor lesions in the bilateral parietal-occipital lobes and right cerebellar hemisphere, accompanied by extensive surrounding cerebral parenchymal edema. Tumor stroke cannot be excluded; metastatic tumors are possible (**Figure 2**).

### Timeline

**Table 1** outlines the critical timeline from symptom onset to treatment.

**Table 1:** The critical timeline from symptom onset to treatment.

2025-06	Symptoms such as headache, blurred vision, and unsteady gait
2025-07-18	CT-guided lung biopsy
2025-07-26	Focal Brain Radiotherapy: Intensity-modulated radiation therapy (IMRT) to the intracranial metastatic lesions, using a large-fraction regimen with a total dose of 52.5 Gy in 15 fractions (3.5 Gy per fraction)
2025-07-31	Albumin-bound paclitaxel (150mg/m², day 1 and day 8) plus carboplatin (area under the plasma concentration-time curve 5, day 1)
2025-08-20	Tislelizumab (200mg/body, day 1) plus albumin-bound paclitaxel (150mg/m², day 1 and day 8), plus carboplatin (area under the plasma concentration-time curve 5, day 1)
2025-09-13	Tislelizumab (200mg/body, day 1) plus albumin-bound paclitaxel (150mg/m², day 1 and day 8), plus carboplatin (area under the plasma concentration-time curve 5, day 1)
2025-10-07	Tislelizumab (200mg/body, day 1) plus albumin-bound paclitaxel (150mg/m², day 1 and day 8), plus carboplatin (area under the plasma concentration-time curve 5, day 1)
2025-10-30	Tislelizumab (200mg/body, day 1) plus albumin-bound paclitaxel (150mg/m², day 1 and day 8), plus carboplatin (area under the plasma concentration-time curve 5, day 1)
2025-11-20	Tislelizumab (200mg/body, day 1) plus albumin-bound paclitaxel (150mg/m², day 1 and day 8), plus carboplatin (area under the plasma concentration-time curve 5, day 1)
2025-12-11	Tislelizumab (200mg/body, day 1)
2026-01-01	Tislelizumab (200mg/body, day 1)
2026-01-22	Tislelizumab (200mg/body, day 1)
2026-02-12	Tislelizumab (200mg/body, day 1)
2025-03-05	Tislelizumab (200mg/body, day 1)

### Diagnostic assessment

**Figure 3:**
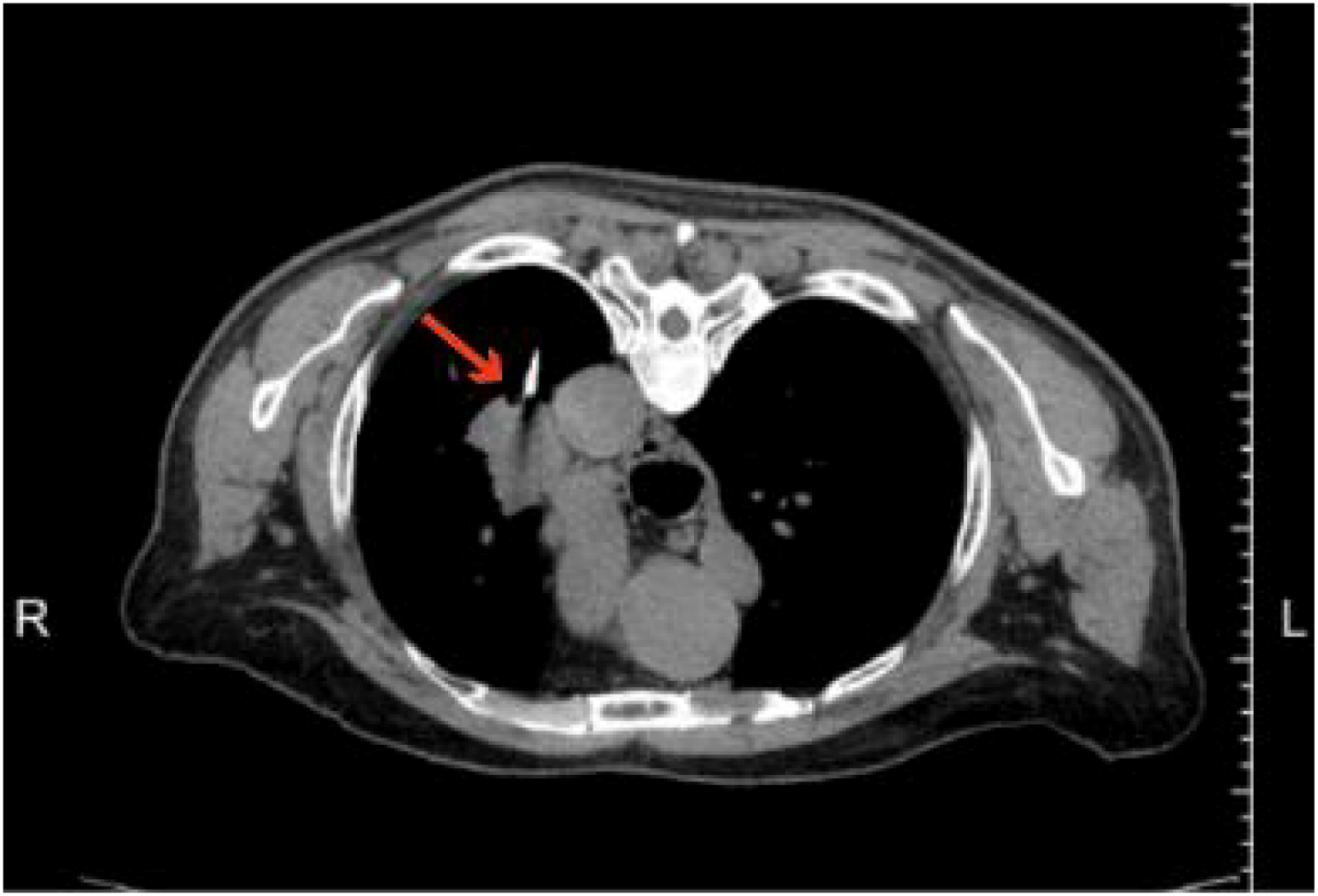
Percutaneous needle biopsy. Under CT guidance, a puncture was performed. The red arrow in figure indicates the puncture needle.

**Figure 4:**
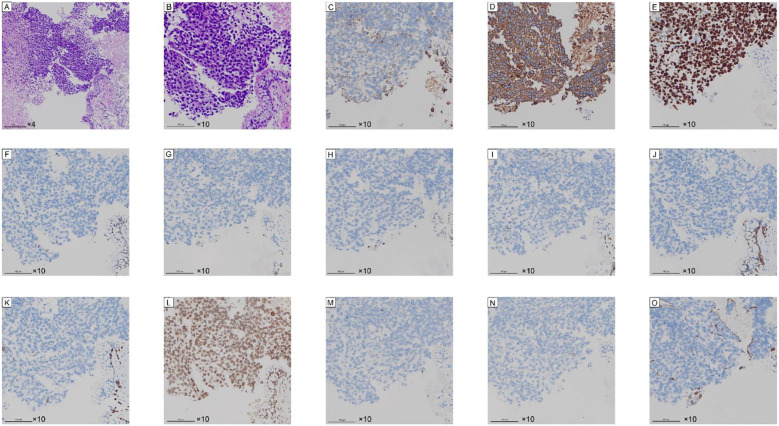
Pathology and Immunohistochemistry. Tumor cells are visible in the center of the image, with a few bronchial epithelial cells noted in the lower right corner. **(A, B)** show H&E staining, revealing tumor cells with large, deeply stained nuclei lacking features characteristic of adenocarcinoma or squamous cell carcinoma. **(C–O)** demonstrate CK7 (focal+); CK (+); Ki-67 (positive rate approximately 70%); SMARCA4 (negative); Napsin A (negative); TTF-1 (negative); P40 (negative); CK5/6 (negative); P63 (negative); INI-1 (positive); CD56 (negative); Syn (negative); Vimentin (negative). **(F)** shows that tumor cells with SMARCA4 gene deletion exhibit BRG1 staining negativity (no staining), while normal nuclei appear brown.

The patient underwent a CT-guided lung biopsy. (**Figure 3**) The histopathology of the puncture tissue showed: (left lung) the puncture tissue showed: malignant tumor with large areas of necrosis, combined with immunohistochemistry, it was consistent with undifferentiated carcinoma of the SMARCA4 deficiency type. Immunohistochemistry: CK7 (focal+); CK (+); Ki-67 (positive rate about 70%); SMARCA4 (-); Napsin A (-); TTF-1 (-); P40 (-); CK5/6 (-); P63 (-); INI-1 (+); CD56 (-); Syn (-); Vimentin (-). (**Figure 4**) The 130 gene detection of lung cancer: TP53 p.N131I mutation, ATM p.F2537L mutation, FANCA p.R1321H mutation; Common driver gene mutations of lung cancer were negative. Microsatellite instability test results: MicroSatellite Stable. No obvious bone metastasis signs were found in the bone scan. Based on pathological and imaging examinations, the patient was definitively diagnosed with SMARCA4-UT, staged as cT2N2M1, stage IV.

### Treatment and efficacy

Following mannitol administration to reduce cerebral edema, the patient underwent cranial metastasis radiotherapy and systemic immunotherapy combined with chemotherapy, followed by maintenance immunotherapy alone.

The patient underwent intensity-modulated radiation therapy (IMRT). Positioned supine, the patient was immobilized using a customized thermoplastic head mask. A simulation CT scan with 2.5 mm slice thickness was performed under intravenous contrast, supplemented by diagnostic MRI for target volume delineation. Specific contouring steps: Draw the entire skull to form the CTV, and extend the CTV outward by 0.5 cm to form the PTV.Bilateral parietal-occipital lobe and right cerebellar hemisphere metastatic lesions were contoured to form the gross tumor volume (GTVm). The planning target volume (PTVg) was defined by extending the GTVm by 8.0 mm. Prescribed dose: PTV:35Gy in 15 fractions, with a daily dose of 3.5 Gy;PTVg:52.5Gy in 15 fractions, with a daily dose of 3.5 Gy. Critical organs included the brainstem, spinal cord, optic nerves, optic chiasm, parotid glands, and temporal lobes. Plan optimization ensured >95% of the GTVm volume received the prescribed dose while strictly limiting doses to all critical organs within QUANTEC-recommended thresholds (e.g., brainstem Dmax ≤ 54 Gy, spinal cord Dmax ≤ 45 Gy). Treatment was delivered using a Varian TrueBeam linear accelerator with image guidance and online positional correction via cone-beam CT prior to each fraction.

Simultaneously with localized cranial radiotherapy, the patient received systemic immunotherapy combined with chemotherapy: Tislelizumab (200mg/body, day 1) plus albumin-bound paclitaxel (150mg/m², day 1 and day 8), plus carboplatin (area under the plasma concentration-time curve 5, day 1). A cycle is 21 days. The patient did not receive tislelizumab during the first treatment because the genetic testing results were not yet available. After 6 cycles of treatment, the patient received regular maintenance therapy with tislelizumab monotherapy. Treatment-related adverse events were graded according to CTCAE 5.0: Grade 1 alopecia, Grade 1 fatigue, Grade 2 anemia (minimum hemoglobin 84 g/L). No Grade ≥3 adverse events were observed.

Patients underwent contrast-enhanced chest CT and contrast-enhanced cranial MRI at baseline and after every two treatment cycles. Tumor response was assessed according to the Response Evaluation Criteria in Solid Tumors (RECIST) version 1.1. The target lesion was selected as the largest, reproducibly measurable lesion among measurable lesions. A maximum of two lesions may be selected per organ, with a total of five lesions selected throughout the body. In this case, the target lesions were: primary lesion of the left pulmonary hilum, hilar lymph node metastasis, left parietal lobe brain metastasis, and right parietal lobe brain metastasis. The sum of the longest diameters of target lesions was 135.5 mm before treatment and 91.0 mm after 4 cycles of treatment, representing a 32.8% reduction in tumor size (>30%). (**Table 2**) This was evaluated as a partial response (PR). Comparison of pre- and post-treatment imaging reveals significant tumor shrinkage with cavitation formation. (**Figures 1**, **2**) The patient’s vision has returned to normal, gait is stable, and ECOG performance status is 0. Each subsequent assessment was Stable Disease (SD) compared to the previous one.

**Table 2:** Longest diameter of target tumor lesions before and after treatment.

Tumor location	Primary lesion of the left pulmonary hilum	Right lung metastasis	Hilar lymph node metastasis	Left parietal lobe brain metastasis	Right parietal lobe brain metastasis	Cerebellar metastatic tumor	Total diameter of target lesions^②^
Pre-treatment diameter (mm)	35.5	14.6	20.0	52.0	28.0	14.0	135.5
Post−four−cycles diameter (mm)	20.0	6.0	11.0	46.0	14.0	5.0	91.0
Reduction ratio^①^	43.7%	58.9%	45.0%	11.5%	50.0%	64.3%	32.8%

^①^Reduction ratio was calculated as: [(Pre-treatment diameter – Post−four−cycles diameter)/Pre-treatment diameter] × 100%.

^②^The target lesions were: primary lesion of the left pulmonary hilum, hilar lymph node metastasis, left parietal lobe brain metastasis, and right parietal lobe brain metastasis.

### Follow-up

The patient’s overall survival and progression-free survival have both exceeded 9 months. The patient’s treatment cycle is 21 days, with imaging follow-up examinations conducted after every two cycles of treatment.

### Patient perspective

The patient expressed great satisfaction with the treatment plan and its efficacy. The current results exceeded his expectations. The restoration of normal vision and mobility, coupled with the absence of significant side effects post-treatment, has greatly enhanced his confidence in the therapy.

## Discussion

SMARCA4 is a tumor suppressor. About 5-7% of human malignant tumors have aberrant SMARCA4 expression (4). The SMARCA4 gene is located on chromosome 19p13. It is a key catalytic subunit of the SWI/SNF complex, and encodes the BRG1 protein. The SWI/SNF complex is a multi-subunit ATP-dependent chromatin remodeling complex. Its main function is to mobilize nucleosomes and reshape chromatin, utilizing the energy released by ATP hydrolysis, hence regulating the transcription of target genes. The SWI/SNF complex is related to the development of cancer, directly interacting with tumor suppressor factors and oncogenes. It may also play a role in DNA synthesis, DNA repair in response to damage, and mitotic gene regulation (5). Loss of SMARCA4 expression has been confirmed to occur in various malignant tumors, such as lung malignancies, digestive tract malignancies, sinus malignancies, uterine and ovarian malignancies, etc (3, 6–9). We report a rare case of thoracic SMARCA4-deficient undifferentiated tumor with multiple brain metastases in an elderly male patient with a history of smoking. Following treatment with cranial metastasis radiotherapy and systemic immunotherapy combined with chemotherapy, the lesions significantly decreased in size, and the patient’s symptoms improved markedly.

### Clinical characteristics

SMARCA4-UT commonly affects young to middle-aged men, with a marked male preference and a history of heavy smoking. The tumor typically presents as a large, invasive mass in the mediastinum, hilum, lungs, and/or pleura. The most common sites of metastasis are lymph nodes, adrenal glands, and bones, while brain metastases are rare (10). SMARCA4-UT is a highly aggressive tumor with a median overall survival of 4 to 7 months (2). The patient in the case report is a 68-year-old male with a history of heavy smoking, consistent with the typical profile of this tumor. The primary lesion was located in the pulmonary hilum, with multiple metastases to the lungs and brain. At the time of diagnosis, the patient’s overall survival was predicted to be within three months. Currently, the patient’s overall survival and progression-free survival have both exceeded 3 months.

### Histology and immunohistochemistry

The histological features of SMARCA4 are epithelioid and rhabdoid tumor cells. Blurred nuclear boundaries and prominent nucleoli are two typical characteristics of the type of tumor cells. These tumor cells typically exhibit extensive necrosis, lack epithelial structure, and grow in non-cohesive clusters. Glandular and squamous differentiation usually does not exist, except in very rare cases when NSCLC is combined. The proliferation rate of Ki-67 has always been very high, approximately 70% (10). In the case report, the patient’s histopathology images reveal tumor cells with large, deeply stained nuclei lacking features of adenocarcinoma or squamous cell carcinoma, with Ki-67 (positive rate about 70%).

### Diagnosis

The clinical symptoms and signs of SMARCA4-UT are not distinct; therefore, SMARCA4-UT must be diagnosed using a mix of laboratory testing, histopathology, and molecular characteristics. The gold standard for the diagnosis of SMARCA4-UT is pathology testing. Some circumstances are usually helpful for the diagnosis of SMARCA4-UT. Pathological features of a striated muscle-like or poorly differentiated phenotype, along with the loss of SMARCA4 expression, are essential criteria. In the results of immunohistochemistry of the patient, the loss of SMARCA4 expression was the most diagnostically significant indicator, while other indicators assisted in differential diagnosis. The distinct immunophenotypes of SMARCA4-UT include complete loss or diffuse severe reduction of SMARCA4 (BRG1) expression, loss of SMARCA2 (BRM) expression, negative or weak/focal expression of cytokeratin and Claudin4. And the frequent expression of stem cell markers (including CD34, SALL4, and SOX2) (11). The patient lacked important biomarker results such as SMARCA2, CD34, SALL4, and SOX2. However, in actual clinical practice, a definitive diagnosis of SMARCA4-UT can be established based on the loss of SMARCA4 expression and differential diagnosis.

### Differential diagnosis

Pathological examination is crucial for the accurate identification of SMARCA4-UT. SMARCA4-UT needs to be differentiated from various thoracic tumors, with a key distinction being its differentiation from SMARCA4-dNSCLC. The former presents with undifferentiated rhabdomyosarcoma-like morphology and often shows loss of epithelial markers (such as Claudin-4), while the latter, although it manifests as adenocarcinoma or squamous cell carcinoma (12). Regarding rhabdomyomorphic and poorly differentiated phenotypes, lack of epithelial structure and strong diffuse keratin expression generally rule out SMARCA4-dNSCLC (13). The final integrated diagnosis requires a combination of histological morphology and immunophenotype, such as using markers such as SOX2 and CD34 to aid in the diagnosis of SMARCA4-UT (14), the loss of Claudin-4 in excluding carcinoma, and negative lymphocyte markers in ruling out diffuse large B-cell lymphoma (3).In the case report, the loss of SMARCA4 expression confirmed that the tumor was SMARCA4-UT, and the lack of adenocarcinoma and squamous cell carcinoma manifestations ruled out NSCLC. Positive results for CK7 and CK definitively confirmed that the tumor was carcinoma, not sarcoma, lymphoma, or melanoma. A negative result for Vimentin further ruled out sarcoma. TTF-1 and Napsin A were highly specific markers for lung adenocarcinoma, and their negative results did not support lung adenocarcinoma. Negative results for squamous cell carcinoma-specific markers P40, P63, and CK5/6 ruled out squamous cell carcinoma of the lung. Negative results for CD56 and Syn ruled out typical neuroendocrine tumors. A positive result for INI-1 indicated normal expression, ruling out other malignant tumors with INI-1 deficiency.

### Current treatment strategy

The risk of early postoperative recurrence is comparatively significant for patients with SMARCA4-deficient tumors, except for stage I patients (15). Obviously, the patient in this case was in an advanced stage and had no chance of surgery.

For patients with advanced SMARCA4-UT disease, immunotherapy combined with chemotherapy or immunotherapy alone is associated with better responses. A retrospective study shows that compared with immunotherapy combined with chemotherapy, the progression-free survival (PFS) of chemotherapy alone was greatly decreased (26.8 months vs 2.73 months, p=0.0437) (1). A retrospective study on SMARCA4-dNSCLC has shown that in adjuvant therapy, the chemosensitivity to platinum-based chemotherapy, such as cisplatin, is higher (16). However, there are currently no studies on the efficacy of chemotherapy alone on SMARCA4-UT. Currently, suggested effective immunotherapy combined with chemotherapy for SMARCA4-UT in case reports includes: tislelizumab combined with etoposide/carboplatin (17), atezolizumab combined with bevacizumab (18), paclitaxel and carboplatin (19), etc. Immune checkpoint inhibitor (ICI) therapy is highly likely to be regarded as an option to treat SMARCA4-deficient tumors with or without PD-L1 IHC response by effectively decreasing the clinical and symptomatic tumor burden (20). Some case reports showed that chemotherapy, immunotherapy, or immunotherapy with ICI as an adjuvant or later treatment may benefit after chemotherapy has failed completely (10). Based on retrospective analysis and case reports, we formulated a systemic immunotherapy combined with chemotherapy regimen for the patient: Tislelizumab (200mg/body, day 1) plus albumin-bound paclitaxel (150mg/m², day 1 and day 8), plus carboplatin (area under the plasma concentration-time curve 5, day 1). A cycle is 21 days. Following the treatment, the patient has achieved a favorable response. Post-treatment imaging revealed that the primary tumor at the hilum had partially cavitated. Cavities form after ischemic necrotic tumor tissue is discharged through the bronchi. Studies have demonstrated that cavity formation may be associated with a better prognosis (21, 22). Considering the gradually worsening side effects of long-term chemotherapy, immunotherapy maintenance therapy was administered after 6 cycles of immunotherapy combined with chemotherapy.

At the initial consultation, the patient presented with severe symptoms attributable to brain metastases. Owing to the blood-brain barrier, achieving effective therapeutic concentrations of drugs within the brain remains challenging. According to the *Chinese Radiotherapy Guidelines for Brain Metastases from Lung Cancer*, for refractory brain metastases—including those with a maximum diameter exceeding 4 cm—the use of hypofractionated stereotactic radiotherapy (HSRT) is recommended. This approach not only ensures local tumor control but also reduces associated adverse effects. The recommended dose regimen is 3.5–4 Gy per fraction, delivered to a total dose of 52.5–60 Gy. Due to limitations in hospital equipment, we are only able to provide intensity-modulated radiation therapy (IMRT), but we are using a high-dose fractionation regimen.

Local radiotherapy plays a crucial synergistic and modulatory role in the implementation and efficacy of systemic immunotherapy combined with chemotherapy regimens. Radiotherapy not only achieves precise local control of the primary tumor site but also induces immunogenic cell death in tumor cells, releases tumor-associated antigens, and activates the local and systemic immune microenvironment. This provides more effective targets for immune checkpoint inhibitors, transforming “cold tumors” into “hot tumors,” thereby improving the response rate to systemic immunotherapy (23). Concurrently, radiotherapy improves local tumor blood supply and alleviates hypoxia, thereby increasing tumor cell sensitivity to chemotherapeutic agents (24). This achieves complementary and synergistic effects between local and systemic therapies without significantly increasing systemic toxicity. In terms of treatment sequencing, the introduction of radiotherapy did not interfere with the standard cycles of immunotherapy combined with chemotherapy, nor did it lead to severe myelosuppression or exacerbation of immune-related adverse reactions. Radiotherapy has an abscopal effect; combination therapy can enhance this effect and significantly improve clinical outcomes (25). This suggests that, in patients with advanced SMARCA4-UT, appropriately administered local radiotherapy can serve as a safe and effective means to enhance the efficacy of immunotherapy combined with chemotherapy. It not only strengthens local control but also helps improve the overall benefits of systemic treatment, providing a reference for comprehensive treatment strategies in similar cases.

### Emerging targeted therapy

The enhancer of zeste homolog 2 (EZH2) is the key catalytic subunit of the multi-comb inhibitory complex 2 (PRC2) to perform functions (26). It has histone methyl transferase (HMT) activity and contributes to the suppression of gene expression by methylating histone H3 at lysine 27. Overexpression of EZH2 is associated with tumorigenesis, invasion, metastasis, and poor prognosis for various tumor types (27). Completed foreign clinical trials have shown that the EZH2 inhibitor Tazemetostat has demonstrated clinical benefits for patients with SMARCA4 gene deletion in lymphoma and solid tumors (28, 29). (**Table 3**) Although SMARCA4-UT was eligible in some clinical trials, no data confirmed its inclusion in the analysis. While Tazemetostat and other EZH2 inhibitors have demonstrated activity in some SMARCB1/SMARCA4-deficient tumors, specific data regarding their efficacy against thoracic SMARCA4-UT remain extremely limited and largely inferred. EZH2 inhibitors represent a promising but still investigational treatment option for SMARCA4-UT. Currently, emerging targeted therapies are not approved and are unavailable in clinical practice. However, with further advancements in clinical trials, new treatment options may become available for patients with SMARCA4-UT in the future.

**Table 3:** Clinical trials of EZH2 inhibitors and SMARCA4-deficient.

Clinical trials	Specify trial number	Trial phase	Trial status	Tumor context	Alteration requirement	SMARCA4-UT patients
Study of the EZH2 Inhibitor Tazemetostat in Malignant Mesothelioma	NCT02860286	II	completed	Mesothelioma	BAP1 Loss of Function	Not eligible
Study of Tazemetostat in Participants With Relapsed or Refractory B-cell Non-Hodgkin’s Lymphoma With EZH2 Gene Mutation	NCT03456726	II	completed	Relapsed or Refractory B-cell Non-Hodgkin’s Lymphoma	EZH2 Gene Mutation	Not eligible
Tazemetostat for the Treatment of Relapsed/Refractory Follicular Lymphoma	NCT05467943	II	Completed	Relapsed/Refractory Follicular Lymphoma With EZH2	EZH2 Gene Mutation	Not eligible
A Study Evaluating CPI-1205 in Patients With B-Cell Lymphomas	NCT02395601	I	Completed	B-Cell Lymphoma	No requirement	Not eligible
EZH2 Inhibitor Tazemetostat in Pediatric Subjects With Relapsed or Refractory INI1-Negative Tumors or Synovial Sarcoma	NCT02601937	I	Completed	Relapsed or Refractory INI1-Negative Tumors or Synovial Sarcoma	No requirement	Eligible
A Study of Tazemetostat in Adult Participants With Soft Tissue Sarcoma	NCT02601950	II	Completed	Soft Tissue Sarcoma includes: Malignant Rhabdoid Tumors, Synovial Sarcoma, INI1-negative Tumors, Epithelioid Sarcoma, any Solid Tumor With an EZH2 GOF Mutation, etc.	No requirement	Not eligible
ORIOn-E: A Study Evaluating CPI-1205 in Patients With Advanced Solid Tumors	NCT03525795	I/II	Completed	Advanced Solid Tumors	No requirement	Eligible
Tazemetostat in Treating Patients With Relapsed or Refractory Advanced Solid Tumors, Non-Hodgkin Lymphoma, or Histiocytic Disorders With EZH2, SMARCB1, or SMARCA4 Gene Mutations (A Pediatric MATCH Treatment Trial)	NCT03213665	II	Completed	Relapsed or Refractory Advanced Solid Tumors, Non-Hodgkin Lymphoma, or Histiocytic Disorders	EZH2, SMARCB1, or SMARCA4 gene mutations	Eligible
Tazemetostat+Nivo/Ipi in INI1-Neg/SMARCA4-Def Tumors	NCT05407441	I/II	Recruiting	INI1-Neg/SMARCA4-Def Tumors	INI-1 (SMARCB1) or SMARCA4	Eligible
A Study of LY4050784 in Participants With Advanced or Metastatic Solid Tumors	NCT06561685	I	Recruiting	Metastatic Solid Tumor, Advanced Solid Tumor, Non-small Cell Lung SMARCA4-Deficient Tumor	BRG1 alteration	Eligible
Tiragolumab and Atezolizumab for the Treatment of Relapsed or Refractory SMARCB1 or SMARCA4 Deficient Tumors	NCT05286801	I/II	Active, not recruiting	Relapsed or Refractory SMARCB1 or SMARCA4 Deficient Tumors	SMARCB1 or SMARCA4 deficient	eligible
Study of Nivolumab and Ipilimumab in Children and Young Adults With INI1-Negative Cancers	NCT04416568	II	Active, not recruiting	INI1 Negative Tumors, other SMARCA4-deficient Malignant Tumors (With PI Approval)	Loss of INI1, loss or mutation of SMARCB1 (INI1), loss or mutation of SMARCA4	Eligible

### Gene mutation

The majority of SMARCA4-UT patients have a history of smoking and typical co-mutations related to smoking. The most frequent SMARCA4-UT co-mutation is TP53, and other frequent co-mutations include CDKN2A, KRAS, STK11, NF1, and PTEN (30). The patient in the case underwent genetic testing, which revealed TP53 p.N131I mutation, ATM p.F2537L mutation, and FANCA p.R1321H mutation. The clinical significance of these mutations in the context of SMARCA4-deficient undifferentiated tumors (SMARCA4-UT) remains incompletely defined. These genes are functionally involved in the DNA damage response pathway, and their alterations may theoretically be associated with homologous recombination repair deficiency, genomic instability, and enhanced sensitivity to DNA-damaging agents such as platinum-based chemotherapy and PARP inhibitors. Currently, all targeted drugs for TP53 mutations are in the clinical research stage globally, and none have been officially approved for marketing. Conventional chemotherapy has limited efficacy in patients with SMARCA4-UT. It is particularly important to deeply analyze its molecular mechanism and real clinical data. Clarifying these mechanisms helps discover their predictive and prognostic biomarkers. Therefore, improving genetic testing will have significant guiding significance for treatment.

## Limitation

SMARCA4-UT is often characterized by a loss of SMARCA2 and the frequent expression of stem cell markers (including CD34, SALL4, and SOX2). However, considering cost and time constraints, not all biomarker tests are performed comprehensively in clinical practice. Priority is given to biomarkers that enable definitive identification. In this case report, the immunohistochemistry results for SMARCA2, CD34, SALL4, and SOX were missing. The patient declined further biomarker testing, limiting our ability to validate the correlation between SMARCA4-UT and SMARCA2 in this case. However, no explicit requirement has been identified in the literature indicating that these factors are necessary for diagnosis.

The absence of data on immune response-related biomarkers, such as PD-L1 expression and tumor mutational burden, represents a limitation of this case. However, several cases have been reported in the literature in which patients with low or negative PD-L1 expression and low tumor mutational burden still derived benefit from immunotherapy (25, 31).

## Conclusion

Given the limitations of current single-drug therapy, the combined use of immunotherapy, chemotherapy, and new target drugs is a choice well worth considering and the future direction. For SMARCA4-UT with brain metastases, radiotherapy plays a critical role in local control and exerts synergistic effects with systemic therapy. Genetic testing holds significant guiding significance for the treatment of SMARCA4-UT. Research into emerging targeted therapies for SMARCA4-UT is urgently needed and promising.

## Data Availability

The original contributions presented in the study are included in the article/supplementary material. Further inquiries can be directed to the corresponding authors.
